# A combined analysis of TyG index, SII index, and SIRI index: positive association with CHD risk and coronary atherosclerosis severity in patients with NAFLD

**DOI:** 10.3389/fendo.2023.1281839

**Published:** 2024-01-08

**Authors:** Wenyuan Dong, Yuxin Gong, Jianqi Zhao, Yanan Wang, Bao Li, Youdong Yang

**Affiliations:** ^1^ Vasculocardiology Department, The Third People’s Hospital of Datong, Datong, Shanxi, China; ^2^ Key Laboratory of Cardiovascular Diseases, Second Hospital of Shanxi Medical University, Taiyuan, Shanxi, China; ^3^ Department of Pathology, Tongji Hospital Affiliated to Tongji Medical College Hust, Wuhan, Hubei, China; ^4^ Vasculocardiology Department, The First People’s Hospital of Jinzhong, Jinzhong, Shanxi, China

**Keywords:** triglyceride-glucose index, systemic immune-inflammation index, systemic inflammation response index, non-alcoholic fatty liver diseases, coronary heart disease

## Abstract

**Background:**

Insulin resistance(IR) and inflammation have been regarded as common potential mechanisms in coronary heart disease (CHD) and non-alcoholic fatty liver disease (NAFLD). Triglyceride-glucose (TyG) index is a novel biomarker of insulin resistance, System immune-inflammation index(SII) and Systemic inflammation response index(SIRI) are novel biomarkers of inflammation, these biomarkers have not been studied in CHD with NAFLD patients. This study investigated the correlation between the TyG index, SII index, and SIRI index and CHD risk among NAFLD patients.

**Methods:**

This cross-sectional study included 407 patients with NAFLD in the Department of Cardiology, The Second Hospital of Shanxi Medical University. Of these, 250 patients with CHD were enrolled in the NAFLD+CHD group and 157 patients without CHD were enrolled as NAFLD control. To balance covariates between groups, 144 patients were selected from each group in a 1:1 ratio based on propensity score matching (PSM). Potential influences were screened using Lasso regression analysis. Univariate and multivariate logistic regression analyses and the Least Absolute Shrinkage and Selection Operator (LASSO) regression were used to assess independent risk and protective factors for CHD. Construction of nomogram using independent risk factors screened by machine learning. The receiver operating characteristic(ROC) curve was used to assess the ability of these independent risk factors to predict coronary heart disease. The relationship between the Gensini score and independent risk factors was reflected using the Sankey diagram.

**Results:**

The LASSO logistic regression analysis and Logistic regression analyses suggest that TyG index (OR, 2.193; 95% CI, 1.242-3.873; *P* = 0.007), SII index (OR, 1.002; 95% CI, 1.001-29 1.003; *P <*0.001), and SIRI index (OR,1.483;95%CI,1.058-2.079,*P*=0.022) are independent risk factors for CHD. At the same time, Neutrophils, TG, and LDL-C were also found to be independent risk factors in patients, HDL-C was a protective factor for CHD in patients with NAFLD. Further analysis using three machine learning algorithms found these independent risk factors to have good predictive value for disease diagnosis, SII index shows the highest predictive value. ROC curve analysis demonstrated that combining the SII index, SIRI index, and TyG index can improve the diagnostic ability of non-alcoholic liver cirrhosis patients with CHD.ROC curve analysis showed that the combined analysis of these independent risk factors improved the predictive value of CHD(AUC: 0.751; 95% CI: 0.704-0.798; *P <*0.001).

**Conclusion:**

TyG index, SII index, and SIRI index are all independent risk factors for CHD in patients with NAFLD and are strongly associated with prediction and the severity of CHD.

## 1 Introduction

Coronary heart disease (CHD) is one of the leading causes of death and disability worldwide, with a common cause being atherosclerosis (1). The formation and development of atherosclerotic plaques is the most important cause of CHD. Traditional risk factors include hypertension, dyslipidemia, smoking, diabetes, genetic predisposition and obesity (2). Recent studies have shown that Insulin resistance and inflammatory responses are also is involved in atherosclerotic plaque formation and remodeling independent of traditional risk factors (3). Despite significant progress in the treatment of CHD, further research is needed on early diagnosis and prevention. As an emerging field, increasing attention is being focused on the connection between IR, systemic inflammation, and the occurrence and development of CHD (4).

Non-alcoholic fatty liver disease (NAFLD) is one of the most common chronic diseases in the world (5). NAFLD has the potential to progress to more serious liver damage such as non-alcoholic steatohepatitis (NASH) (6).NASH is characterized by inflammatory changes that can lead to progressive liver damage, cirrhosis, and hepatocellular carcinoma (HCC) (7). Meanwhile, NAFLD is also a major risk factor for cardiovascular disease. An increasing number of studies have shown that NAFLD and CHD share common risk factors. Recent studies have shown that in addition to obesity, hypertension, dyslipidemia, diabetes, and genetic predisposition, Insulin resistance and inflammatory responses also play an important role in cardiovascular events in patients with NAFLD (8). Triglyceride-glucose index (TyG index) is a reliable indicator for the diagnosis of IR because of it performs better than the high insulin-glucose clamp test (9). SII index and SIRI index are two novel indicators of inflammation and immunity, that have been proven to be associated with the risks for CVDs and all-cause mortality (10), SII index was non-linear associated with all-cause mortality in individuals with NAFLD (11). A population-based study imply that the SII index and TyG index are related to NASH (12). however, the relationship between the SIRI index and CHD in NAFLD patients remains unclear. This study aims to analyze the potential associations between the TyG index, SII index, and SIRI index, and the occurrence as well as the severity of CHD in NAFLD patients.

## 2 Materials and methods

### 2.1 Experimental design and participant inclusion

This study is a retrospective study that included a total of 407 patients with NAFLD. These patients were divided into two groups, the non-alcoholic fatty liver disease group (NAFLD group) and the non-alcoholic fatty liver disease combined with coronary heart disease group (NAFLD+CHD group), coronary angiography was done at the Second Hospital of Shanxi Medical University. For patients with CHD, the Gensini score was further used to evaluate the severity of coronary stenosis (13). The diagnosis of NAFLD is based on the Asia-Pacific Working Party recommendations (14). Firstly, other factors that can cause fatty liver, such as excessive alcohol consumption, a history of viral hepatitis, and the use of hepatotoxic drugs, are excluded. Ultrasound technicians evaluate using standard methods (15).However, we cannot yet further confirm the presence of NASH patients among NAFLD patients because of the lack of liver biopsy results in the study. Additionally, this study excluded other potential confounding factors including congenital heart disease, a history of coronary heart disease, oral lipid-lowering medications, autoimmune diseases, malignancies, the use of anti-platelet drugs, quality of diabetes regulation, and patients with cerebrovascular accidents. This study has been approved by the Ethics Committee of Shanxi Medical University Second Hospital and all participating patients have signed informed consent forms.

### 2.2 Data collection and computation

This study retrospectively collected the basic information and laboratory indicators of enrolled patients from the electronic medical record system of the Second Hospital of Shanxi Medical University. The basic information includes gender, age, height, weight, history of hypertension (systolic and diastolic blood pressure), history of diabetes, coronary angiography report, smoking history, alcohol consumption history, and medication use. The body mass index (BMI), Triglyceride glucose product index (TyG), Systemic immune-inflammation index (SII), and systemic inflammation response index (SIRI) are calculated as follows: BMI = W(kg)/H(m)2, TyG = Ln[TG(mg/dL)×FBG(mg/dL)/2] (16); SII = (Plt×N/L) (17); SIRI = (N×M/L) (18).

### 2.3 Machine learning

This study used four machine learning methods to select variables and assess their importance in diagnosis and prognosis. Lasso regression analysis was used to address confounding factors that could potentially affect the results. Based on the potential influencing factors, the random forest model (RF), support vector machine model (SVM), and generalized linear model (GLM) were constructed using the ‘ caret ‘ R software package (version 6.0-86). Seven potential influences were used as explanatory variables and CHD as a response variable (19). The three models obtained were analyzed using the “explain” function in the’DALEX’ R package, and the cumulative residual distributions were plotted. The cumulative residual distribution was plotted to determine the best predictive model.RF, GLM, and SVM were further utilized to analyze the significance of various risk factors in disease occurrence. All these procedures were conducted using R (version 4.3.1).

### 2.4 Propensity score matching analysis

Propensity score matching analysis (PSM) is a recognized method for eliminating the influence of potential confounding factors, and after PSM adjustment, data can be considered free from interference by these confounding factors. In non-alcoholic fatty liver disease patients, this study used Lasso regression to obtain confounding factors (including BMI, lymphocytes, monocytes, serum creatinine, and total cholesterol) as independent variables to establish a model with the occurrence of CHD as the dependent variable. The caliper value of the model was set at 0.02. After adjusting for these confounding factors, the validity of PSM was assessed using quartiles and absolute standard deviations. The results showed a good balance between the CHD+NAFLD and NAFLD groups after performing 1:1 PSM matching.

### 2.5 Statistical analysis

Statistical analysis software IBM SPSS (version 23.0) and GraphPad Prism (version 8.0) were used to analyze the data in this study. Continuous variables were described using mean ± standard deviation (SD) or median (IQR), while categorical variables were expressed as percentages. The Shapiro-Wilk test was employed to assess the normality of continuous variables, and the independent sample t-test or Mann-Whitney test was used for between-group comparisons of continuous variables. Between-group comparisons of categorical variables were conducted using the chi-square test or Fisher’s exact test.

### 2.6 Receiver operating characteristic

First, Lasso regression analysis was used to screen confounding factors, followed by Propensity Score Matching (PSM) to correct for these confounders. Logistic regression analysis was then performed using the corrected data to further identify potential risk factors for CHD in NAFLD patients. Multivariate analysis was conducted to determine independent factors that can influence the occurrence of the disease. A nomogram was constructed using the selected risk factors, and receiver operating characteristic (ROC) curve analysis and area under the curve (AUC) values were used to evaluate their predictive ability for the disease (20). Additionally, Spearman correlation analysis was employed to assess the relationship between Gensini scores and several independent risk factors.

## 3 Results

### 3.1 Baseline characteristics of the NAFLD and NAFLD+CHD groups

This study collected a total of 407 patients with NAFLD, among whom 250 patients had CHD. Among these patients, the average age was 59 years for those with NAFLD-CHD and 55 years for those with NAFLD alone. As shown in **Table 1**, there were no significant differences in BMI, platelet count(Plt), lymphocyte count(L), and total cholesterol levels(TC) between the two groups. However, the proportion of male patients, with hypertension, diabetes mellitus, and smokers was higher in the NAFLD+CHD group (p*<*0.05). Additionally, neutrophil count(N), triglyceride level(TG), low-density lipoprotein level(LDL-C) and fasting blood glucose(Glu) level were higher in the NAFLD+CHD group compared to the NAFLD group (p*<*0.05), while lymphocyte count and high-density lipoprotein level were lower in the NAFLD+CHD group compared to the NAFLD group (p*<*0.05).

**Table 1:** Demographic and clinical characteristics of participants by the presence of CHD.

Characteristics	CHD +NAFLD (n=250)	NAFLD (n=157)	P
Age (years)	59 (51, 67)	55 (48, 62)	< 0.001
Sex (male)	176 (43.2%)	81 (19.9%)	< 0.001
BMI (kg/m^2^)	25.96 (24.77, 27.775)	25.82 (24.16, 27.72)	0.429
HT [n (%)]	169 (41.5%)	73 (17.9%)	< 0.001
DM [n (%)]	96 (23.6%)	19 (4.7%)	< 0.001
Smoke [n (%)]	134 (32.9%)	49 (12%)	< 0.001
Plt (10^9^/L)	216.5 (177.25, 258.25)	225 (195, 258)	0.117
N (10^9^/L)	4.335 (3.4325, 5.9375)	3.48 (2.73, 4.61)	< 0.001
L (10^9^/L)	1.84 (1.4525, 2.33)	1.94 (1.58, 2.37)	0.105
M (10^9^/L)	0.475 (0.37, 0.6175)	0.47 (0.34, 0.54)	0.048
Cr (mmol/L)	67 (58.25, 76)	65 (56, 74)	0.027
TC (mmol/L)	4.4689 ± 1.1113	4.3691 ± 0.98557	0.358
TG (mmol/L)	1.805 (1.335, 2.6)	1.61 (1.19, 2.2)	0.001
HDL-C (mmol/L)	1.05 (0.91, 1.23)	1.17 (1, 1.34)	< 0.001
LDL-C (mmol/L)	2.485 (2.075, 2.975)	2.27 (1.78, 2.71)	< 0.001
Glu (mmol/L)	5.86 (4.9925, 7.2375)	5.3 (4.94, 5.77)	< 0.001
TyG	9.1 (8.76, 9.5275)	8.84 (8.53, 9.16)	< 0.001
SII	519.44 (356.27, 770.47)	410.04 (304.5, 558.74)	< 0.001
SIRI	1.1191 (0.71674, 1.7797)	0.81013 (0.51493, 1.1947)	< 0.001

All the above data are expressed as mean ± standard deviation or median (quartiles). The Chi-square test was used for categorical variables.Paired and unpaired t-tests were used for comparison of normal values, and Wilcoxon rank sum test or Mann-Whitney test was used for comparison of non-normally distributed values. p<0.05 was considered statistically significant.

BMI, body mass index; HT, hypertension; DM, diabetes mellitus; Plt, platelet; N, neutrophil; L, lymphocyte; M, monocyte; Cr serum, creatinine; TC, total cholesterol; TG, triglycerides; HDL-C, high-density lipoprotein cholesterol; LDL-C, low-density lipoprotein cholesterol; Glu, fasting plasma glucose; TyG, triglyceride-glucose index; SII, immune-Inflammation Index; SIRI, systemic inflammation response index.

### 3.2 Lasso regression screening for risk factors

**Figure 1:**
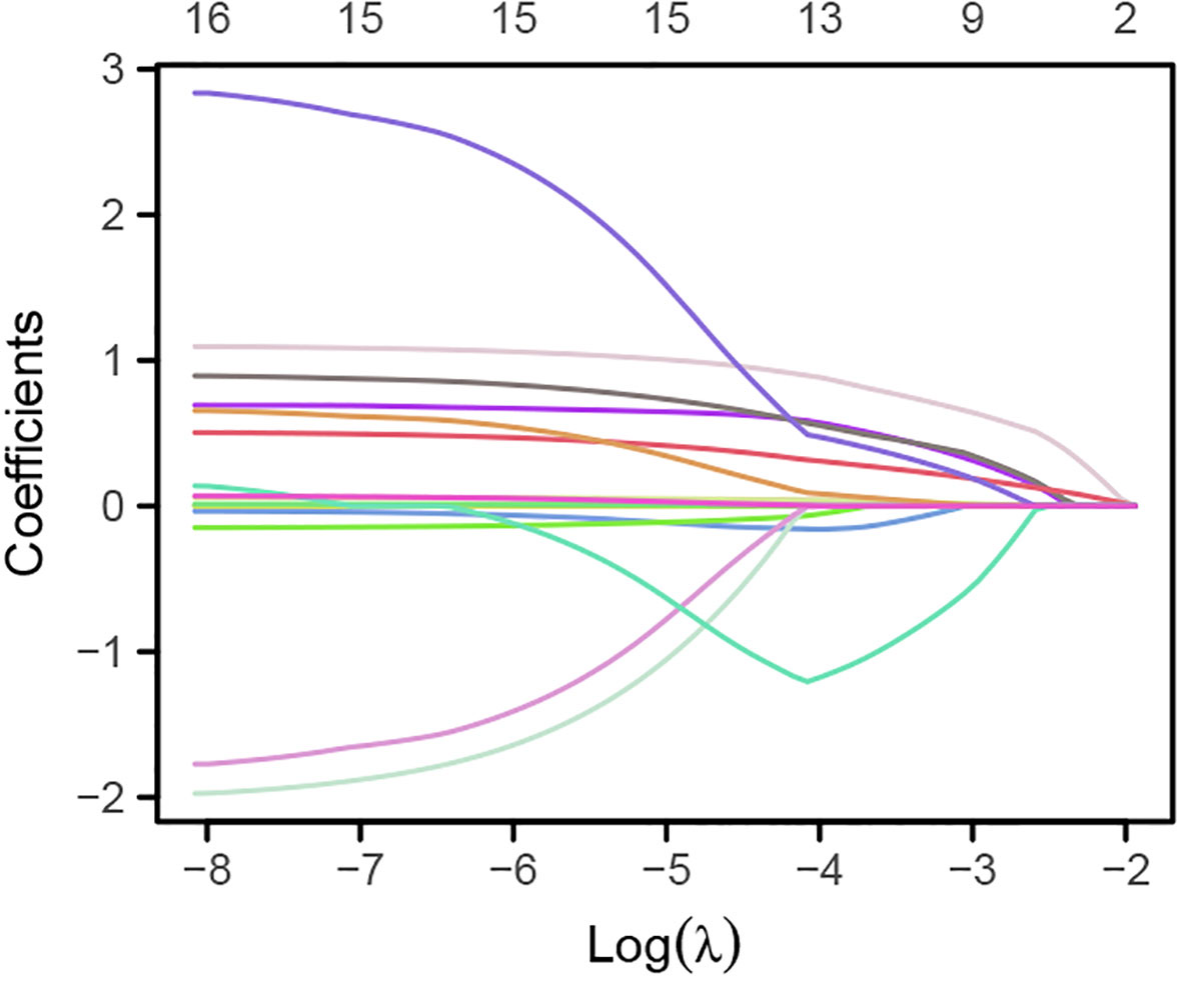
Variation of the 16 variables with the regularization parameter *λ*.

**Figure 2:**
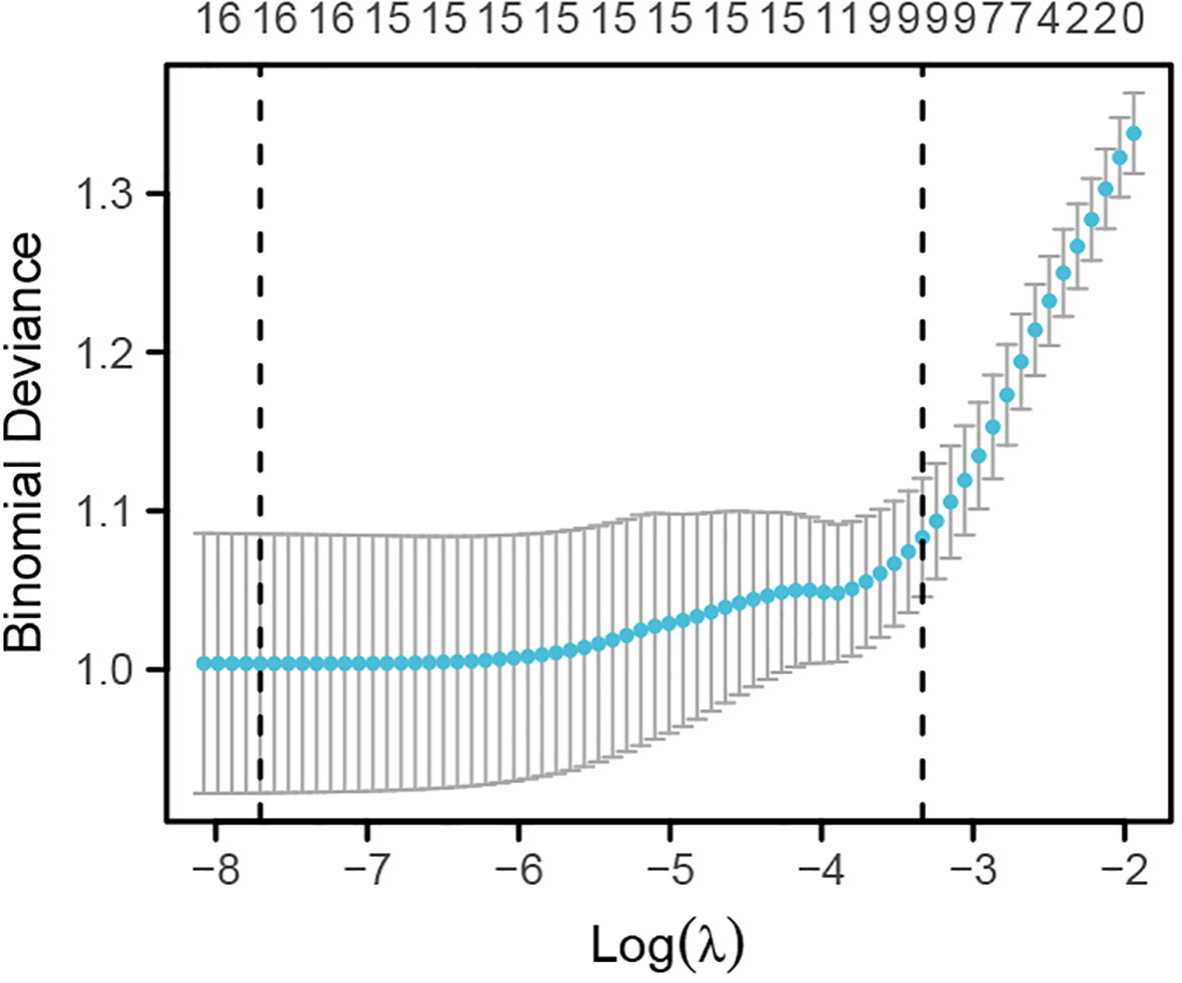
Lasso regression analysis of the optimal regularization parameter *λ*.

Lasso regression analysis was used to identify potential risk factors for CHD from the aforementioned 16 variables. The results, as shown in **Figures 1**, **2**, revealed that BMI, platelet count, lymphocytes, monocytes, creatinine, total cholesterol, and fasting random blood glucose were excluded from consideration as these seven indicators did not significantly impact CHD. The remaining nine indicators were considered potential risk factors for CHD (**Supplementary Table 1**).

### 3.3 Propensity score matching analysis

Propensity score matching analyses were then performed on the uncorrelated factors identified by the Lasso regression analyses to eliminate any potential effect of these confounding variables on the results. The results, as shown in **Table 2**, revealed that after propensity score matching, there were no statistically significant differences (p*>*0.05) between groups in terms of BMI, platelet count, lymphocyte count, monocyte count, creatinine level, total cholesterol level, and fasting random blood glucose. Additionally, after propensity score matching, there were also no statistically significant differences (p*>*0.05) between groups in terms of gender and smoking history.

**Table 2:** Demographic and clinical characteristics of participants after PSM analysis.

Characteristics	CHD+NAFLD (n=144)	NAFLD (n=144)	P
BMI (kg/m^2^)	26 (24.22, 27.76)	25.995 (24.385, 28.04)	0.850
Plt (10^9^/L)	223.5 (183.75, 268)	224 (188.75, 258)	0.887
L (10^9^/L)	1.885 (1.4775, 2.33)	1.935 (1.595, 2.3625)	0.355
M (10^9^/L)	0.43 (0.35, 0.5325)	0.475 (0.3675, 0.5525)	0.286
Cr (mmol/L)	64 (54, 72)	66 (56, 75)	0.449
TC (mmol/L)	4.3429 ± 1.0494	4.369 ± 0.98124	0.828
Glu (mmol/L)	5.345 (4.86, 6.2825)	5.365 (4.95, 5.8325)	0.626
Sex (male)	90 (31.2%)	79 (27.4%)	0.188
Age (years)	59 (51, 66)	54.5 (48, 62)	0.006
HT [n (%)]	93 (32.3%)	66 (22.9%)	0.001
DM [n (%)]	33 (11.5%)	17 (5.9%)	0.013
Smoke, n (%)	64 (22.2%)	48 (16.7%)	0.053
N (10^9^/L)	4.085 (3.2675, 5.1725)	3.54 (2.7275, 4.78)	< 0.001
TG (mmol/L)	1.74 (1.3175, 2.3825)	1.595 (1.17, 2.2)	0.015
HDL-C (mmol/L)	1.1072 ± 0.23081	1.1859 ± 0.26976	0.008
LDL-C (mmol/L)	2.41 (2.0075, 2.8925)	2.265 (1.78, 2.715)	0.030
TyG	8.95 (8.67, 9.27)	8.835 (8.53, 9.155)	0.012
SII	502.45 (326.84, 680.06)	417.51 (316.18, 560.56)	0.006
SIRI	0.93323 (0.63843, 1.4049)	0.8698 (0.56531, 1.2157)	0.045

All the above data are expressed as mean ± standard deviation or median (quartiles). The Chi-square test was used for categorical variables.Paired and unpaired t-tests were used for comparison of normal values, and Wilcoxon rank sum test or Mann-Whitney test was used for comparison of non-normally distributed values. p<0.05 was considered statistically significant.

BMI, body mass index; HT, hypertension; DM, diabetes mellitus; Plt, platelet; N, neutrophil; L, lymphocyte; M, monocyte; Cr serum, creatinine; TC, total cholesterol; TG, triglycerides; HDL-C, high-density lipoprotein cholesterol; LDL-C, low-density lipoprotein cholesterol; Glu, fasting plasma glucose; TyG, triglyceride-glucose index; SII, immune-Inflammation Index; SIRI, systemic inflammation response index.

### 3.4 Univariate and multivariate analysis of CHD-related factors in NAFLD

**Figure 3:**
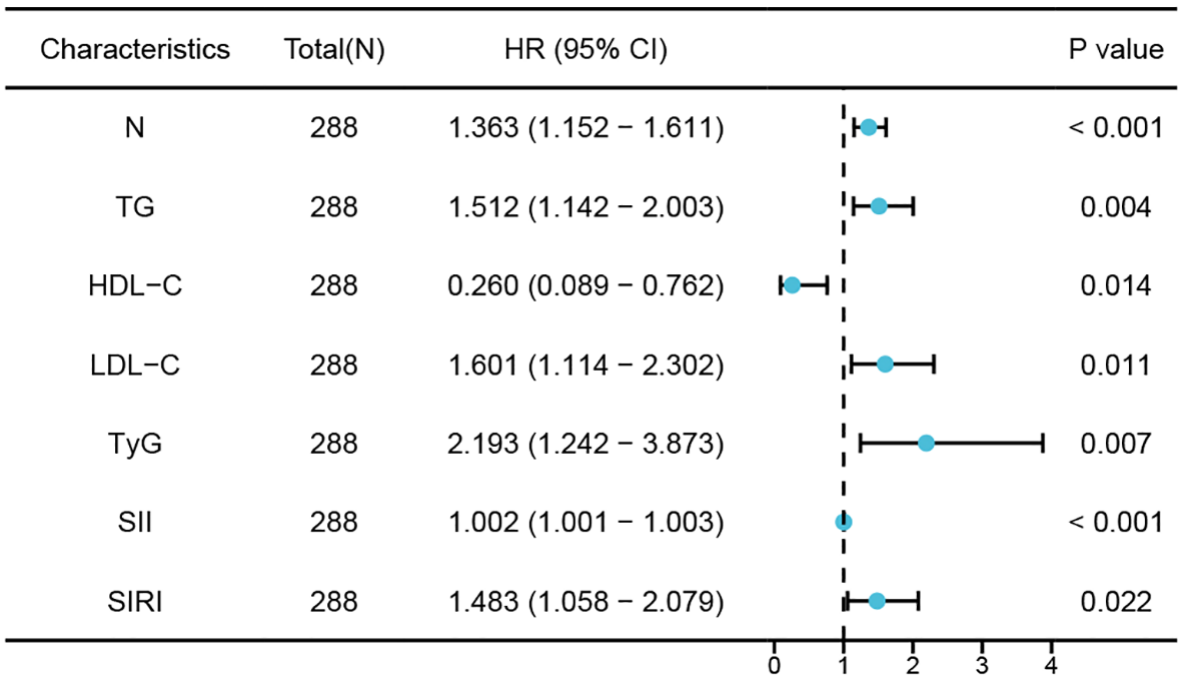
Forest plots of independent factors associated with CHD in NAFLD.

Univariate logistic regression analysis showed significant correlations between neutrophils, TG, HDL-C, LDL-C, TyG index, SII index, and SIRI index with the occurrence of CHD in NAFLD patients. Furthermore, multivariate logistic regression analysis revealed that neutrophils (OR 1.363; 95% CI 1.152 - 1.611; *P <*0.001), TG (OR 1.512; 95% CI 1.142 - 2.003; *P* =0.004), LDL-C (OR 1.601; 95% CI 1.114 -2.302; *P* =0.011), TyG index (OR,2.193;95%CI,1242-3.873;*P*=0.007),SII index(OR,1002;95%CI,10011-1003;*P<*0.001)and SIRI index(OR,1483;95%CI,1058-2079;*P*=002) remained are independent risk factors for NAFLD patients developing CHD while HDL-C (OR,0260;95%CI,0089-0762;p=014) was still an independent protective factor where elevated levels of HDL-C reduce risk of CHD in NAFLD patients (**Table 3**; **Figure 3**).

**Table 3:** Univariate and multivariate analyses of factors associated with CHD and NAFLD.

Characteristics	Total(n)	Univariate analysis		Multivariate analysis (Model I)		Multivariate analysis (Model II)	
		Odds Ratio (95% CI)	P value	Odds Ratio (95% CI)	P value	Odds Ratio (95% CI)	P value
N	288	1.339 (1.139 - 1.575)	< 0.001	1.413 (1.196 - 1.670)	< 0.001	1.363 (1.152 - 1.611)	< 0.001
TG (mmol/L)	288	1.414 (1.091 - 1.832)	0.009	1.518 (1.156 - 1.994)	0.003	1.512 (1.142 - 2.003)	0.004
HDL-C (mmol/L)	288	0.283 (0.109 - 0.733)	0.009	0.185 (0.067 - 0.510)	0.001	0.260 (0.089 - 0.762)	0.014
LDL-C (mmol/L)	288	1.431 (1.013 - 2.023)	0.042	1.460 (1.029 - 2.070)	0.034	1.601 (1.114 - 2.302)	0.011
TyG	288	2.111 (1.257 - 3.546)	0.005	2.226 (1.313 - 3.775)	0.003	2.193 (1.242 - 3.873)	0.007
SII	288	1.002 (1.001 - 1.003)	< 0.001	1.002 (1.001 - 1.003)	< 0.001	1.002 (1.001 - 1.003)	< 0.001
SIRI	288	1.527 (1.092 - 2.137)	0.013	1.602 (1.137 - 2.257)	0.007	1.483 (1.058 - 2.079)	0.022

Model I was adjusted for age and sex. Model II was adjusted for age, sex,hypertension, diabetes mellitus and smoking history.

### 3.5 Machine learning models evaluate biomarkers

**Figure 4:**
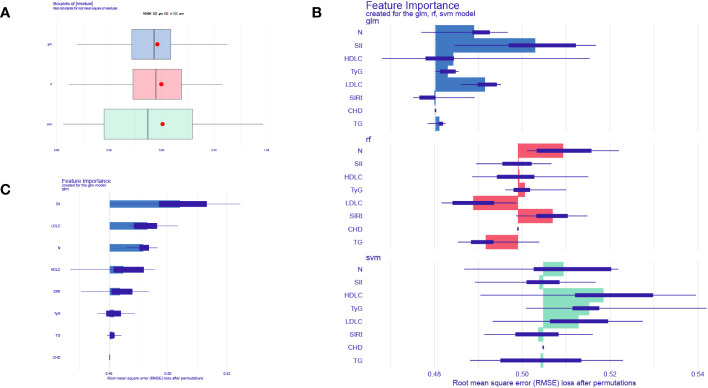
Machine learning models evaluate biomarkers. **(A)** Boxplots of the three machine learning models. **(B)** Three machine learning models to assess the importance of seven factors for CHD. **(C)** GLM model showing the importance of 7 factors for CHD prediction.

To further validate the accuracy of these risk factors in predicting the occurrence of CHD in NAFLD patients, this study used three machine learning algorithms to evaluate the importance of seven predictive factors in disease diagnosis. We chose Random Forest (RF), Support Vector Machine (SVM), and Generalize linear model(GLM) as our test models (21). We used the DALEX package to explore, interpret, and evaluate the models. The optimal model is selected for the next analysis based on the residual values. As shown in **Figure 4A**, the boxplot represents the accuracy of CHD prediction by the three machine.

learning models, with the GLM model having the smallest residual and thus indicating the highest accuracy. **Figure 4B** illustrates the importance of CHD prediction by the three machine learning models, while **Figure 4C** displays the importance of each factor in CHD prediction within the GLM model. The results demonstrate that all seven influencing factors have significant value in predicting CHD, with SII index, LDL-C, neutrophils(N), HDL-C, SIRI index, TyG index, and TG being sequentially ranked according to their predictive importance.

### 3.6 Establishing a diagnostic nomogram for CHD in patients with NAFLD

**Figure 5:**
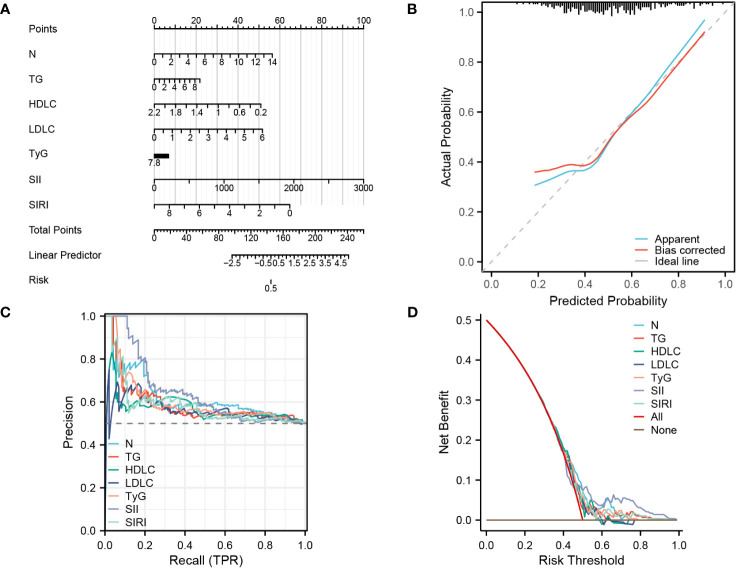
Establishing a diagnostic nomogram for CHD in patients with NAFLD **(A)** nomogram for the diagnosis of CHD. **(B)** Calibration curve for prediction accuracy. **(C)**, PR curve for diagnosis of CHD. **(D)**, DCA curve for the diagnosis of CHD.

Through univariate and multivariate logistic regression analysis, as well as validation using three machine learning models, this study discovered that seven factors have significant potential for diagnosing and predicting CHD. Therefore, this study utilized these seven factors to construct a clinical nomogram model.

(**Figure 5A**). The calibration curve in **Figure 5B** demonstrates that the actual risk aligns closely with the predicted risk after bias correction, indicating a high level of consistency between them. Additionally, in the diagnostic PR curve, all factors except LDL-C exhibit higher predictive ability than the baseline prediction line, indicating a high level of accuracy in these predictions (**Figure 5C**). As shown in **Figure 5D**, the predictive curves for all factors are mostly above both the ALL curve and None curve, suggesting a substantial net benefit for patients.

### 3.7 ROC analysis predicts factors

**Figure 6:**
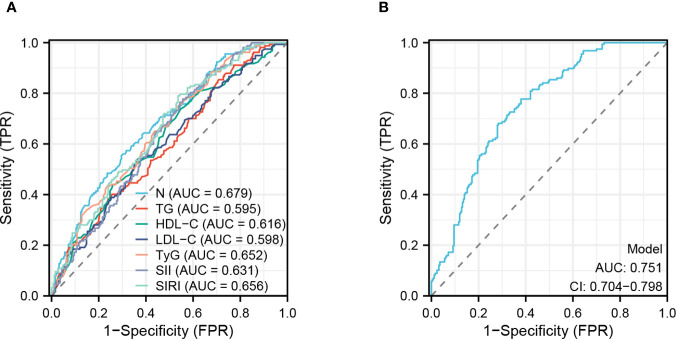
ROC analyses of risk factor. **(A)** Analysis of subject work characteristics (ROC) curves for the predictive ability of each risk factor for CHD. **(B)** Overall prediction curves for the combined individual factors.

This study evaluated the accuracy of various predictive factors in predicting the occurrence of CHD in NAFLD patients using ROC curves (**Figure 6A**). The results showed that N (AUC = 0.679), TG (AUC = 0.595), HDL-C (AUC = 0.616), LDL-C (AUC = 0.598), TyG (AUC = 0.652), SII (AUC = 0.631), and SIRI (AUC = 0.656) had different levels of accuracy in prediction. Furthermore, this study assessed the diagnostic accuracy of all factors combined (**Figure 6B**) and found a significant improvement in predictive accuracy when using a combination of these seven predictive factors, with an AUC: 0.751 (CI: 0.704-0.798; *P<*0.001).

### 3.8 Association between predictors and coronary Gensini score

**Figure 7:**
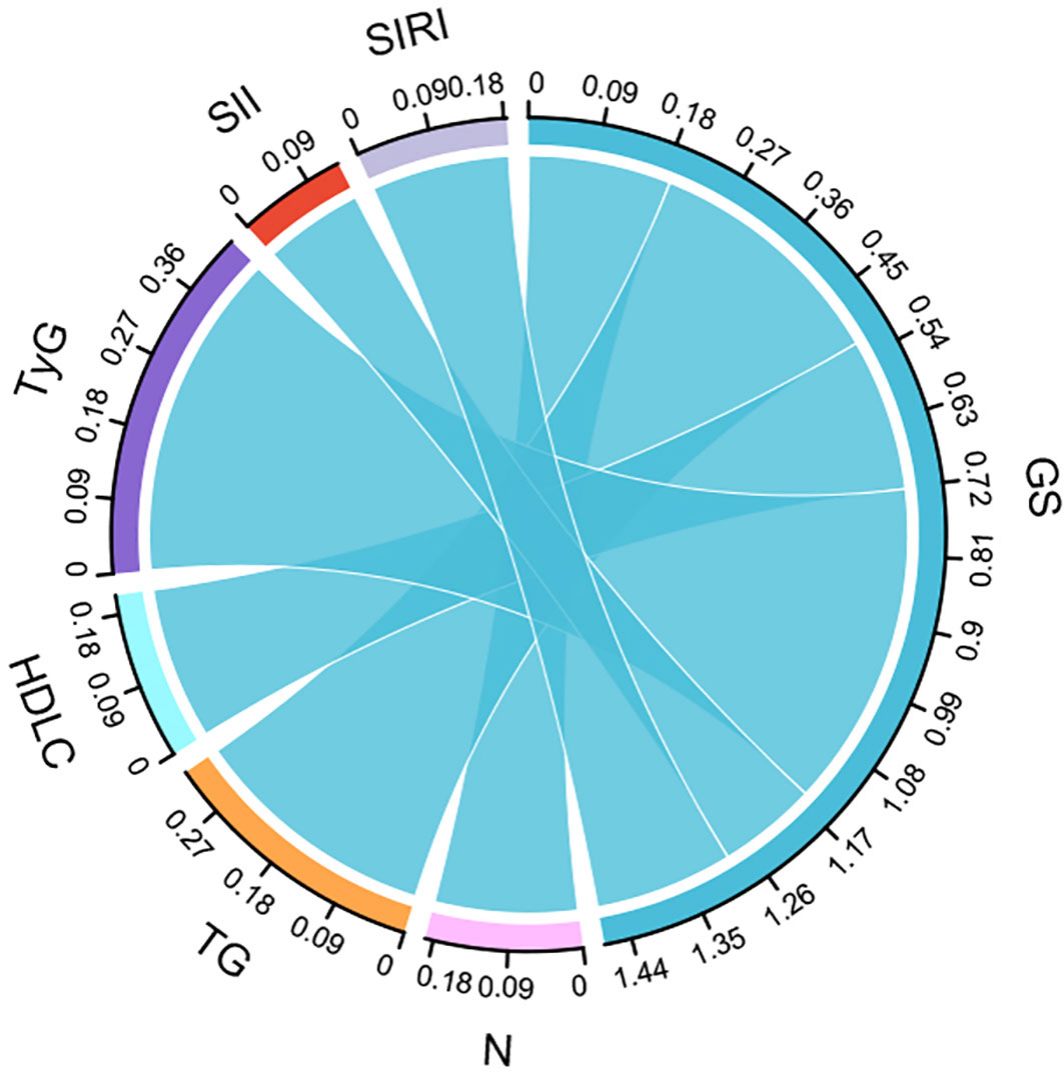
Sankey diagram reflecting the correlation between factors and CHD severity.

This study used Spearman’s correlation analysis to examine the relationship between Gensini score and neutrophils, TG, HDL-C, LDL-C, TyG, SII, and SIRI. The results showed no significant correlation between the Gensini score and LDL-C (r=0.082,*P*=0.121).The Gensini score was weakly correlated with neutrophils (r=0.19, *P<*0.001), TG (r=0.329, *P<*0.001), HDL-C (r=-0.203, *P<*0.001), SII (r=0.132, *P*=0.004), and SIRI (r=0.184, *P*= 0.025) but strongly correlated with TyG (r= 0.435,*P <*0001) (**Figure 7**).

## 4 Discussion

NAFLD is an inflammation-associated metabolic disease, with steatosis as its most prominent feature. NAFLD is a continuum of disease with or without mild inflammation (non-alcoholic fatty liver) to non-alcoholic steatohepatitis (NASH) (5). NASH is characterised by a more rapid progression of necroinflammation and fibrosis than non-alcoholic fatty liver. NAFLD patients included in this study include non-alcoholic fatty liver and non-alcoholic steatohepatitis. Cardiovascular disease is one of the leading causes of death in NAFLD patients, and NAFLD patients are more likely to develop atherosclerosis, cardiovascular disease, and arrhythmias (22, 23). CHD is prevalent in patients with NAFLD, NAFLD is considered a high-risk group for CHD (8). NAFLD and CHD share many common pathogenic mechanisms, Many studies have suggested that IR and inflammation are underlying mechanisms common to both NAFLD and CHD.

IR is a systemic disease affecting multiple organs throughout the body, which makes it play an important role in the onset and progression of NAFLD and CHD (24). The cardiometabolic syndrome(CMS) is typically characterised by IR, IR may have an effect on cardiovascular disease through decreases in nitric oxide (NO) and increases in vascular stiffness (25). At the same time, IR is closely related to NAFLD, and IR is involved in various liver diseases such as steatosis, NASH and liver fibrosis (26). Patients with NAFLD have impaired liver function, resulting in an inability to suppress the production of glucose and fatty acids, Localised lipid and glucose accumulation leads to the onset and development of IR, IR leads to systemic disorders of glucose metabolism and lipid metabolism, ultimately leading to damage to the vascular endothelium (27), This explains why NAFLD is a high risk group for CHD. The TyG index is a reliable indicator of IR,A clinical study enrolling 62,443 Chinese individuals suggests that the TyG index is an independent risk factor for cardiovascular disease and may help identify individuals potentially at high cardiovascular risk (28). Another clinical study, which lasted follow-up 10 years and included 6,095 CVD patients with undiagnosed diabetes mellitus, demonstrated that the TyG index can be used as a clinical.

predictor of CVD and CHD in the non-diabetic population (29). This suggests that TyG index can still be an independent risk factor for CHD in low-risk populations. However, there are still fewer studies on the TyG index in populations at high risk of CHD. TyG index also has good diagnostic value in NAFL,A cross-sectional study including 10,761 patients suggests that the TyG index is effective in identifying people at risk of NAFLD (30). Wang et al. showed that TyG index was independently and positively associated with NAFLD in the general population (31). And another cohort study showed that TyG index showed good diagnostic performance for NASH in obese patients. Thus, TyG index is an independent risk factor for both NAFLD and CHD.

Systemic inflammation plays an important role in CHD and NAFLD development. Systemic inflammation is directly related to the development of atherosclerosis, it is well known that coronary atherosclerosis is the main cause of CHD, and immune cells and systemic inflammation play an important role in the accumulation of lipids in the matrix of intima of coronary arteries (2). Some studies have shown that patients with systemic inflammatory diseases have a much higher risk of CVD than the general population (32). Similarly, systemic inflammation can lead to IR, and promotes the development of NAFLD. By analysing 47 high-quality serum samples from patients with NAFLD, Haukeland et al. found that mild systemic inflammation was prevalent in patients with NAFLD and that CCL2 (chemokine (C-C motif) ligand 2) and MCP1 (monocyte chemoattractant protein) may play a key role in the disease (33). Lipids accumulation in hepatocytes leads to local inflammation and immune cell activation. On the one hand, activated immune cells cause systemic inflammation by releasing pro-inflammatory cytokines and chemokines (34). On the other hand, activation and recruitment of hepatic immune cells via signals from adipose tissue may promote inflammatory responses leading to cell injury and death, thereby promoting NAFLD disease progression (35). In summary, NAFLD can promote CHD through IR and systemic inflammation. NAFLD is not only associated with morbidity and mortality from liver disease, but is also an important risk factor for the development and progression of CHD. SII index and SIRI index are two novel systemic inflammatory biomarkers, A 20-Year Follow-Up Cohort Study of 42,875 US Adults found that high SII or SIRI increased adverse cardiovascular outcomes in the general population (36). Another cohort study has also shown that the SII index can be a useful marker for predicting the development of cardiovascular disease in middle-aged and older adults (37). Clinical studies have shown that inflammation is associated with the progression of NAFLD, A cross-sectional study of 6,792 adult Chinese on fatty liver showed a significant positive correlation between SII index and hepatic steatosis (17), NAFLD is an inflammation-related metabolic disease, and hepatic steatosis is the most prominent manifestation of the disease. Given that both IR and systemic inflammation lead to an increased risk of NAFLD with CVD and that NAFLD is a high-risk factor for CHD, both the TyG index, SII index, and SIRI index can predict NAFLD and CVD. There are no studies that have simultaneously explored the effects of IR and systemic inflammation on CHD in patients with NAFLD. Therefore, this work aims to explore this association.

Our findings suggest that TyG index, SII index, and SIRI index are all independent risk factors for CHD in patients with NAFLD. Neutrophils, TG, and LDL-C were also screened as independent risk factors for CHD by using three machine learning algorithms, while HDL-C was an independent protective factor for CHD, Of these, TG, LDL-C and HDL-C are all traditional independent risk factors for CHD (38), Interestingly, Neutrophil thought to cause CHD in NAFLD patients, There is still a lack of clarity regarding the role of neutrophil in NAFLD and CHD, Emerging evidence suggests that neutrophils are important players in several chronic diseases, such as atherosclerosis, nonalcoholic fatty liver disease and autoimmune disorders (39, 40). Neutrophils are normally the first responders to acute inflammation and contribute to the resolution of inflammation, However, in chronic inflammation neutrophils may promote inflammation through different mechanisms such as neutrophil accumulation within the tissue, protease release and NET formation (41). In addition, ROC analyses showed that TyG index (AUC = 0.652),SII index (AUC = 0.631),and SIRI index (AUC = 0.656) had average predictive power for CHD occurrence, this may be related to the sample size included in the study. By constructing a nomogram, this study designed a clinical diagnostic model for predicting CHD in patients with NAFLD, ROC analysis shows good diagnostic power for CHD on combined analysis of 7 independent risk factors (AUC: 0.751;CI: 0.704-0.798; *P <*0.001).

This study also has some limitations. Firstly, this is a retrospective study with a limited sample size included. Second, because of the lack of liver biopsy results, no distinction was made between NAFLD and NASH at the time of patient inclusion. Finally, because this was a cross-sectional study, the results showed that the TyG index, SII index, and SIRI index was positively associated with the occurrence of CHD in patients with NAFLD, but it was not able to determine whether it had predictive value. Although this study attempted to construct a diagnostic model to predict CHD, a large number of large-scale, multicentre prospective studies will be needed in the future to illustrate further the predictive value of these independent risk factors for the occurrence of CHD in patients with NAFLD.

## 5 Conclusion

This study found that in the NAFLD patient population, patients with concurrent CHD had significantly elevated levels of TyG index, SII index, and SIRI index in their serum. Furthermore, the TyG index, SII index, and SIRI index were positively correlated with the severity of CHD. This study also constructed a nomogram to help predict the occurrence of CHD by incorporating abnormal lipid metabolism and inflammation levels into the diagnostic system. Due to their simplicity, convenience, and non-invasive nature, these indicators have great value in routine clinical diagnosis as they can help identify high-risk cardiovascular patients within the NAFLD patient population. However, further research is needed to determine their clinical diagnostic value.

## Data availability statement

The original contributions presented in the study are included in the article/**Supplementary Material**. Further inquiries can be directed to the corresponding author.

## Ethics statement

The studies involving humans were approved by Ethics Committee of the Second Hospital of Shanxi Medical University; Second hosptial of Shanxi medical university. The studies were conducted in accordance with the local legislation and institutional requirements. The participants provided their written informed consent to participate in this study.

## Author contributions

WD: Writing – original draft. YG: Formal Analysis, Writing – original draft. JZ: Data curation, Investigation, Writing – original draft. YW: Software, Writing – review & editing. BL: Writing – review & editing. YY: Writing – review & editing.
