# Supplementary material for: A combined analysis of TyG index, SII index, and SIRI index: positive association with CHD risk and coronary atherosclerosis severity in patients with NAFLD
Source: Front Endocrinol (Lausanne). 2024 Jan 8;14:1281839. doi: 10.3389/fendo.2023.1281839 (PMC10802119; doi:10.3389/fendo.2023.1281839)
Supplement: Supplementary file 1 [file DataSheet_1.doc]

list = ls())

library(tidyverse)

library(caret)

library(DALEX)

setwd("dlexStety <- read.table(file = "input.txt",s = 1)

set.seed(123)lity<-as_tibble(airquality)

airquality<-airquality%>%

drop_na()

airquality

id<-sample(2,nrow(airquality),replace = T,prob = c(0.7,0.3))

airqualitytrain<-airquality[id == 1,]

airqualitytest<-airquality[id == 2,]

ozone_rf<-train(CHD~.,data = airqualitytrain,

method = "rf",

ntree = 500)

ozone_glm<-train(CHD~.,data = airqualitytrain,

method = "glm")

ozone_svm<-train(CHD~.,data = airqualitytrain,

method="svmRadial")

explainer_rf<-explain(ozone_rf,label = "rf",

data = airqualitytest,

y = airqualitytest$CHD)

explainer_glm<-explain(ozone_glm,label = "glm",

data = airqualitytest,

y = airqualitytest$CHD)

explainer_svm<-explain(ozone_svm,label = "svm",

data = airqualitytest,

y = airqualitytest$CHD)

ner_rf)

per_glm<-model_performance(explainer_glm)

per_svm<-model_performance(explainer_svm)

plot(per_rf,per_glm,per_svm,geo = "boxplot")

importance_glm<-variable_importance(

explainer_glm,

loss_function = loss_root_mean_square

)

importance_svm<-variable_importance(

explainer_svm,

loss_function = loss_root_mean_square

)

plot(importance_rf,importance_glm,importance_svm)

#importance_gbm<-variable_importance(explainer_gbm,loss_,lnefunction = loss_root_mean_square

)

plot(importance_svm)

importance_rf<-variable_importance(

explainer_rf,

loss_function = loss_root_mean_square

)

plot(importance_rf)

importance_glm<-variable_importance(

explainer_glm,

loss_function = loss_root_mean_square

)

plot(importance_glm)
